# Semiquantitative Analysis in PET/CT Imaging of Prostate Cancer

**DOI:** 10.3390/jcm14113828

**Published:** 2025-05-29

**Authors:** Vasiliki Fragkiadaki, Ioannis Ntanasis-Stathopoulos, Michalis Liontos, Flora Zagouri, Meletios-Athanasios Dimopoulos, Maria Gavriatopoulou

**Affiliations:** 1 Department of Clinical Therapeutics, School of Medicine, National and Kapodistrian University of Athens, 11528 Athens, Greece; vasiafrag@gmail.com (V.F.);; 2 Current address: Department of Medicine, Korea University, Seoul 02841, Republic of Korea

**Keywords:** semiquantitative analysis, prostate cancer, positron emission tomography, standard uptake value maximum, metabolic tumor volume

## Abstract

Prostate cancer is the second most common cancer, affecting millions of men globally and having a significant burden on health care systems. During recent years, the rapid development of the nuclear medicine field and the wide use and application of positron emission tomography combined with computed tomography (PET/CT) have significantly changed the diagnosis, treatment approach and patient outcomes. Semiquantitative analysis in PET/CT imaging quantifies the load of the disease in patients diagnosed with prostate cancer without measuring the precise amount of a radiotracer injected into the patient; instead, there is an indirect evaluation of the radiotracer using semiquantitative indices. Beginning with the standard uptake value (SUV) in Fluorodeoxyglucose (FDG) PET/CT, various semiquantitative measures have been created and are now used for analyzing different radiotracers. The purpose of this review is to provide an overview of the importance of the semiquantitative analysis in PET/CT imaging with the use of prostate-specific radiotracers at the initial staging of prostate cancer, as well as in biochemical recurrence and in the metastatic state.

## 1. Introduction

According to global cancer statistics, prostate cancer is one of the most common malignancies among men [1] and the second leading cause of malignancy-related mortality after lung cancer in the United States [2]. Great progress has been made since 2011 regarding the risk stratification of prostate cancer patients and in optimizing the therapeutic strategies [3]. Chemotherapy, androgen receptor pathway inhibitors (ARPis), PARP inhibitors (PARPi), radioligand therapy (RLT) and immunotherapy are some of the available therapeutic options not only in castration-resistant prostate cancer but also in the castration-sensitive disease, offering evidence of overall survival benefit [4].

In most of the oncological studies, PET has not been used to evaluate patients, and it has spread rapidly during recent years in clinical practice without adequate data for its proper use. In prostate cancer imaging, PET/CT has become an essential modality [5,6].

EAU-EANM-ESTRO-ESUR-ISUP-SIOG Guidelines on Prostate Cancer have included PSMA PET/CT both at the initial stage of the disease and in the biochemical recurrence and in response to treatment evaluation. Overall, the guidelines recommend PSMA PET imaging during the primary diagnosis/initial staging of the disease for patients with intermediate and high-risk prostate cancer and for those with recurrence after initial treatment [7].

Furthermore, PSMA PET/CT imaging is a prerequisite before starting radioligand therapy. Baseline uptake on prostate-specific membrane antigen (PSMA)-targeted imaging is a prerequisite for treatment with [X^177^Lu]Lu-PSMA-617 [8].

**Figure 1:**
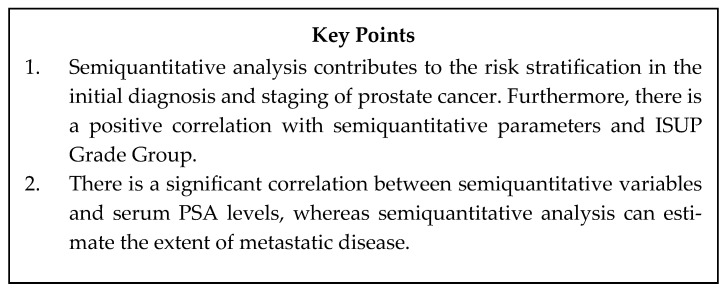
Key points of the review.

The purpose of this review is to highlight the contribution of the semiquantitative analysis used in PET CT imaging to the estimation of the load of prostate cancer and the proper staging of the disease (Figure 1).

## 2. Materials and Methods

In this narrative review, we conducted a thorough search for available published studies between 1 January 2022 and 28 February 2025 in order to focus on the most updated and complete studies. We conducted the search using the PubMed and Google Scholar databases. The following search keywords and headings were used: “Prostate cancer”, “Semiquantitative analysis”, “Positron Emission Tomography”, “PET/CT”, “Standard uptake value maximum” and “Metabolic tumor volume”. We did not include duplicate studies or irrelevant studies and abstracts, or studies that were not written in the English language. Overall, we included 10 eligible studies.

## 3. Background

### 3.1. The Use of PET/CT in Prostate Cancer Imaging

The conventional imaging of prostate cancer includes multiparametric magnetic resonance imaging (mpMRI), with diffusion weighted (DW) and dynamic contrast enhanced (DCE) imaging, mainly for local staging. However, the use of MRI and computed tomography (CT) for locoregional nodal disease detection is limited, due to limitations in size thresholds regarding the short axis diameter of the lymph nodes that can be infiltrated by the disease (8–10 mm) and they may appear normal [9]. At the same time, bone scintigraphy (BS), which is essential for evaluating metastatic bone disease, works best when PSA levels are high, and is less effective when PSA levels are low [10]. The introduction of positron emission tomography (PET) radiopharmaceuticals overcame the deficiencies of the conventional imaging.

### 3.2. The Role of Different Radiopharmaceuticals in Prostate Cancer PET/CT Imaging

Today, the most frequently used PET radiotracers for imaging prostate cancer are those that include the prostate-specific membrane antigen (PSMA) choline or fluciclovine. ^18^F-fluorodeoxyglucose (FDG), which is commonly used for other cancers, is not the first choice for diagnosing or staging prostate cancer. Its role is limited in poorly differentiated neoplasms, such as neuroendocrine and small cell histological subtypes [11] (Table 1).

Furthermore, there is no malignancy in all focal [^18^F]FDG uptake, and incidental uptake is most of the time inflammatory. In cases of biochemical recurrence, the usefulness of [^18^F]FDG PET/CT goes up in Grade Groups 4 or 5 and when PSA levels are high. Its use is also under active research for theragnostic approaches in prostate cancer, including radioligand therapy with [177Lu]Lu-PSMA. Dual tracer staging using FDG and PSMA imaging improves the staging of the disease. Specifically, the addition of [^18^F]FDG PET/CT imaging allows for the evaluation of discordant disease (PSMA negative/FDG positive). Discordant [^18^F]FDG/PSMA uptake suggests that these patients may derive less benefit from [177Lu]Lu-PSMA therapy. In summary, [^18^F]FDG PET/CT imaging is important for advanced prostate cancer and PSMA-negative disease because it helps predict outcomes and is useful for new targeted treatment options [12].

The radiotracers targeting the prostate-specific membrane antigen (PSMA) have gained widespread use in PET/CT imaging of prostate cancer. The first ^68^Ga-labelled PSMA radiopharmaceutical was approved by the Food and Drug Administration (FDA) during 2020. PSMA is a membrane antigen that is overexpressed in prostate cancer cells. Choline participates in the cell membrane synthesis. Many studies have already proven the superiority of PSMA over choline tracers to detect the metastatic disease [13,14,15,16,17,18]. Numerous PSMA-based tracers are currently available; however, there is a lack of consensus on the optimal radiotracer for PSMA PET/CT.

[^18^F]DCFPyL, [^18^F]PSMA-1007, [^18^F]JK-PSMA-7, [^18^F]rhPSMA-7 and [^18^F]AlF-PSMA-11 are the main 18F-based radiotracers. [^68^Ga]Ga-PSMA-11 is the most commonly used ^68^Ga based radiotracer. ^18^F-based radiotracers offer a higher image resolution as they have lower end-point positron energy and longer half-life. The production of ^68^Ga is more challenging, as it requires an on-site generator, and the transportation of ^68^Ga from another site is difficult due to its short half-life, making cheaper the production of [^18^F]PSMA-1007. On the other hand, owing to their shorter half-life, ^68^Ga -based PSMA radiotracers result in lower radiation exposure [19,20].

Furthermore, [^18^F]PSMA-1007 leads to more false positive findings due to higher benign bone uptake but has a greater locoregional lesion detection rate and accuracy in the delineation of the local lesion [21,22,23,24]. This is the result of greater lesion SUV uptake and a predominant hepatobiliary excretion route [25]. [^18^F]DCFPyL has a similar biodistribution as [^68^Ga]Ga-PSMA-11 and a similar bladder uptake. Furthermore, [^18^F]DCFPyL is not associated with increased coeliac ganglia uptake [26,27].

In the current literature, [^18^F]JK-PSMA-7, [^18^F]rhPSMA-7 and [^18^F]AlF-PSMA-11 all demonstrated marginally greater detection rates in comparison with [^68^Ga]PSMA-11. The sensitivity of [^18^F]JK-PSMA-7 lies in its ability to detect more lesions in small anatomic structures [28]. [^18^F]rhPSMA-7 is more effective at detecting lesions adjacent to the bladder [29,30].

## 4. Semiquantitative Analysis and PET/CT

Visual analysis is the primary way of assessing the metabolically active lesion. Nevertheless, qualitative, semiquantitative and absolute quantitative methods are three major categories of PET image evaluation. The most subjective method of the three of them is the qualitative assessment. However, this method is the most frequently used technique in clinical practice. Semiquantitative analysis includes certain semiquantitative indices like the Standardized Uptake Value (SUV) and its variants like SUVmax, SUVmean, SUVlean and target-to-background ratio. Absolute quantitative parameters include mathematical models, Patlak–Gjedde graphical analysis and non-linear regression models. Absolute quantification of the radiopharmaceutical that is concentrated in each lesion is not technically possible or practical for everyday clinical practice. Semiquantitative analysis in PET/CT imaging has a variety of clinical applications, including prostate cancer staging and the assessment of tumor response to therapy. Furthermore, it estimates the metastatic potential as well as the risk of relapse of the disease and facilitates the selection of the optimal site for biopsy, contributing to radiation therapy planning [31].

### Standard Uptake Value (SUV)

The most widely used semiquantitative variable in PET/CT imaging is the standard uptake value (SUV) and particularly its maximum (SUVmax).

The SUV measurement in the tissue or in the lesion is a semiquantitative approach reflecting the concentration of the radiotracer at a certain time point. It is the ratio of the concentration of the radioactivity of the radiopharmaceutical to body weight (tissue tracer activity/[injected dose/patient weight]), and it is a relative index of uptake of the radiopharmaceutical in a two-dimensional or volumetric region of interest (ROI). The measured activity in the tissue is normalized to the average radioactivity according to patient’s body weight (SUVbw), lean body mass (SUL) or body surface area (SUVbsa). This normalization prevents confusing factors like fat tissue from interfering. The SUV is reported either as the maximum (SUVmax) or as the mean (SUVmean) value of all voxels within a specific ROI. The average amount of radiotracer activity in a region of interest (SUVpeak) looks at the average uptake of radiotracer in the area around the voxel with the highest intensity. It decreases image noise and preserves the reproducibility of SUVmax. When SUVpeak is normalized to lean body mass, it is referred as peak SUL (SULpeak) [32,33].

Another relevant variable is the metabolic tumor volume (MTV), which is defined as the volume inside a defined ROI that encompasses the metabolically active tumor. Total metabolic tumor volume (TMTV) is the sum of the separate volumes that we measure from each lesion. Total lesion glycolysis (TLG) is the output of the mean SUV and the MTV. PSMA-TV and ΤL-PSMA are the same parameters as MTV and TLG but for imaging with PSMA radiotracers [34].

## 5. Clinical Applications

Table 2 summarizes the main characteristics and outcomes of the included studies. The discussion of the studies is structured in two main sections: primary diagnosis/initial staging of prostate cancer and biochemical recurrence/metastatic prostate cancer.

### 5.1. Primary Diagnosis and Initial Staging of Prostate Cancer

Dong, S. et al. retrospectively evaluated 60 patients with recently diagnosed localized prostate cancer. All patients were divided into low-intermediate-risk (LIR) or high-risk (HR) groups. Patients in the LIR group had PSA levels ≤ 20 ng/mL, a Gleason score < 8 and clinical stage cT1-cT2c. Patients in the HR group were required to meet at least one of the following criteria: PSA > 20 ng/mL, Gleason score 8–10 or clinical stage ≥ cT3a. The patients underwent ^18^F-PSMA-1007 PET/CT and they reported that the semiquantitative analysis of the primary tumor on ^18^F-PSMA-1007 PET/CT imaging contributes to the risk stratification of prostate cancer, while the semiquantitative variable TL-PSMAp was better to determine high-risk prostate cancer [35].

Furthermore, Yi, N. et al. used SUVmax, as it was calculated in the ^68^Ga-PSMA PET/CT images of 147 patients with localized prostate cancer, to prove that it was a predicting factor of intermediate and high-risk prostate cancer. The PSA levels ranged from 1.23 to 2790 ng/mL. It was reported that high-risk patients and patients in the International Society of Urological Pathology ISUP grade group 3 (GG3) had higher values of median SUVmax with the specificity and the positive predictive value of high-risk prostate cancer patients being, respectively, 95% and 96% with a cut-off SUVmax value of 10.12 [36].

The importance of SUVmax as a predicting factor in ^68^Ga -PSMA PET/CT imaging was also proven by Heetman et al. Specifically, researchers created logistic regression models for 386 patients with International Society of Urological Pathology (ISUP) Grade Group (GG)  ≥  2 and GG  ≥  3 prostate cancer, using noninvasive information collected before a biopsy, such as age, prostate-specific antigen density, presence of a PI-RADS 5 lesion, signs of cancer spreading outside the prostate on MRI and SUVmax of the prostate in ^68^Ga -PSMA PET/CT. Models with and without SUVmax were compared using Likelihood ratio tests and the area under the curve (AUC). DeLong’s test was used to compare the AUCs. They concluded that SUVmax ameliorates diagnostic accuracy in predicting the likelihood of clinically significant prostate cancer in biopsy material [37].

The conclusion from both of the above-mentioned studies is that the early detection of high risk for recurrence or metastatic prostate cancer not only contributes to better treatment planning but is also necessary in order to avoid overtreatment.

In addition to the above, regarding the intraprostatic SUVmax, Rogic, I. et al. observed a positive correlation with the ISUP group among 34 prostate cancer patients who underwent [^68^Ga]Ga-PSMA-11 PET/CT. The inclusion criteria of the study were histologically proven primary prostate cancer, no treatment before PET/CT procedure and a PSA value measured within one month before the scan. The mean PSA value was 33.8 ± 40.9 nmol/L (range 2.2–232). The patients were retrospectively evaluated, with high-risk patients having higher SUVmax values than the low-risk patients, as the PSMA expression was proportionally increased with the Gleason score. This means there is evidence that supports using [^68^Ga]Ga-PSMA-11 PET/CT for the first assessment of prostate cancer [38].

Above, we focused on the usefulness of the semiquantitative analysis and pathological and histopathological parameters. But what about semiquantitative variables and their correlation with biochemical parameters? An answer to this question came from the cross-sectional study of Ali, H. et al. In their analysis, they included patients with histopathologically proven adenocarcinoma of the prostate with organ-confined disease. The PSA level was obtained within 6 weeks before the Galium-68 PSMA PET-CT, and the patients had not received any treatment before the PET/CT imaging. The mean and median PSA levels were 32.33 ng/mL (range: 0.004–306.00) and 14.20 ng/mL, respectively. They found that not only was the SUVmax correlated with the Gleason score and prostate-specific antigen (PSA) in organ-confined prostate cancer, but also the median SUVmax and PSA directly related to the Gleason score in a total of 154 patients. The Gleason score of all patients ranged from 6 to 10 while the mean and median PSA levels were 32.33 ng/mL and 14.20 ng/mL respectively. Of particular importance was that SUVmax was higher in patients with a PSA level of more than 10 than those with a value below 10 [39].

The retrospective analysis of Andela, S.B. et al. aimed to investigate the importance of quantitative imaging parameters of PSMA PET/CT including not only the SUVmax but also tumor asphericity (ASP), PSMA tumor volume (PSMA-TV) and PSMA total lesion uptake (PSMA-TLU). They included 86 patients with localized intermediate- or high-risk PCA and PSMA-PET before treatment. The mean PSA was 11.6 (2.55–130.5). Cox regression analyses were performed for biochemical recurrence-free survival, overall survival (OS), local control and loco-regional control (LRC). Semiquantitative analysis revealed a significant association of PSMA-TV (*p* = 0.003), PSMA-TLU (*p* = 0.004) and ASP (*p* < 0.001) with overall survival (OS) [40]. Bodar, Y.J.L. et al. conducted a bi-centric secondary analysis of two prospective cohort studies which included 318 patients with histologically proven prostate cancer before robotic-assisted radical prostatectomy (RARP). All patients received a PSMA-PET/CT before RARP, of whom 288/318 (91%) underwent ePLND. Included patients had a median initial PSA level of 10.4 (7.2–19.8) ng/mL. According to EAU guidelines, 76/318 (23.9%) patients had intermediate-risk PCa and 242/318 (76%) high-risk PCa. They proved that SUVmax was correlated with pISUP score and pathological tumor stage. Lower SUVmax values were seen in patients with a pISUP of ≤2 compared to patients with a pISUP of >2 for 18 F-PSMA as well as 68 Ga-PSMA-11 (SUVmax18 F-PSMA: median 5.1 vs. 9.6, *p* = 0.002 and SUVmax68 Ga-PSMA-11: 6.6 vs. 8.6, *p* = 0.003) [41].

### 5.2. Biochemical Recurrence and Metastatic Prostate Cancer

The definition of biochemically recurrent prostate cancer according to American Urological Association/American Society for Radiation Oncology/Society of Urologic Oncology guidelines is a rise in the levels of PSA in the blood after radical treatment like surgery or radiation therapy (PSA of 0.2 ng/mL and a confirmatory value of 0.2 ng/mL or greater after surgery and a nadir of + 2.0 ng/mL after radiation therapy) [44].

Metastatic prostate cancer includes de novo and metachronous prostate cancer, two entities that have different patient and tumor characteristics, with different prognosis and overall survival [45]. Τhe 5-year survival rate of patients with metastatic prostate cancer is around 30% [46]. The group of those patients is highly heterogenous. While many patients will have recurrence after local treatment, around 5% of them will present with de novo metastatic disease. The variation also concerns metastatic lesions, the burden of the disease, functional status and cancer-related symptoms. Despite the fact that androgen deprivation therapy (ADT) remains the pillar of treatment of metastatic hormone-sensitive prostate cancer, chemotherapy (docetaxel, cabazitaxel) and oral anti-androgens have proved to have survival benefit when added to ADT [47,48]. Novel treatment options like androgen receptor pathway inhibitors (ARPIs), PARP and PD1 inhibitors as well as radioligand therapy with Lu177 and Ra223 and their combinations are also available, and it has already been proven that they improve the overall survival [49].

Fragkiadaki, V. et al. prospectively investigated 104 patients with biochemically recurrent prostate cancer after radical therapy (with surgery plus radiotherapy or radiotherapy alone) who underwent ^18^F-PSMA-1007 and ^18^F-choline PET/CT imaging. The average PSA of the 80 patients who had metastatic disease in the analysis was 3.79 (±6.18) ng/mL and the median PSA was 1.89 ng/mL., ranging from 0.01 to 40.91 ng/mL. They proved that there is a positive correlation between PSA levels and the semiquantitative parameters SUVmax and TMTV of the metastatic foci measured with both radiotracers [33].

The importance of the findings of this study is that PSA, which is the main tumor marker of prostate cancer, is correlated with the semiquantitative parameters of the PET/CT imaging. Consequently, with higher serum PSA values there is a greater risk of the lesions found in PET/CT imaging being metastatic rather than benign. Hence, the PSA value is a marker of the tumor load and provides an estimation of the burden of the disease, whereas the sensitivity of PSMA and choline PET/CT to detect metastasis increases with higher PSA levels.

Cardoza-Ochoa et al. calculated via specific software the total lesion prostate-specific membrane antigen (wbTl-PSMA) and whole-body PSMA-derived tumor volume (wbPSMA-TV) of each metastatic foci on ^18^F-PSMA-1007 PET/CT in 110 patients with biochemically recurrent prostate cancer. The Spearman analysis proved a statistically significant correlation of volumetric imaging parameters with serum PSA levels and a significant correlation was found between wbTL-PSMA (R = 0.63, *p* < 0.0001) and wbPSMA-TV (R = 0.49, *p* < 0.0001) with serum PSA. A statistically significant difference with wbTL-PSMA was found in patients with a PSA less than or equal to 0.5 ng/mL and PSA in the range of 0.51–1.0 ng/mL. This conclusion is of additive value to the current knowledge regarding the importance of the semiquantitative analysis and represents a useful tool for clinicians to assess the burden of the disease [42].

In the retrospective analysis of Li, Y. et al., 110 patients were divided into three groups, one group with newly diagnosed non-metastatic, another with oligometastatic and the third with extensively metastatic disease. AUCs for the Gleason score (GS), total prostate-specific antigen (TPSA), SUVmax, TL-PSMAp and PSMA-TVp were 0.851, 0.916, 0.834, 0.938, and 0.923, respectively. GS, TPSA, SUVmax, TL-PSMAp and PSMA-TVp were significantly different among the groups. The results of this study revealed that TL-PSMAp and PSMA-TVp could distinguish between oligometastatic and extensive metastatic disease, while GS, TPSA and SUVmax did not. In this analysis, ^18^F-PSMA-1007 PET/CT parameters PSMA-TVp and TL-PSMAp could predict oligometastatic disease [43].

## 6. Limitations

Our study has several limitations. Firstly, in neuroendocrine differentiated prostate cancer PSMA expression is often reduced or lost, so PSMA PET/CT is not a useful imaging modality in those cases. Moreover, while semiquantitative analysis is a useful tool for clinicians, further validation and standardization are needed before routine integration into clinical staging. Moreover, some of the aforementioned studies are retrospective with a small sample size; thus, the generalization of their results should be made with caution. Larger randomized clinical trials are needed in order to reach firm conclusions. Furthermore, the elevated values of PSA do not always correlate with elevated SUV values, as there are discordant cases, both in the literature and in the clinical practice. Therefore, it is important to use PET/CT in conjunction with other imaging modalities such as CT and MRI, along with a clinical examination. Finally, in PET/CT imaging there are lesions that are small in size with low radiotracer avidity but they may represent foci of active disease that necessitate further treatment. However, in the era of biomarker-driven therapies, PSMA PET/CT is particularly useful in the triage of patients with metastatic prostate cancer and helps in tailoring treatment options.

## 7. Conclusions

The induction of prostate specific radiotracers was a revolution in the imaging of prostate cancer. The ability to evaluate how the radiopharmaceutical is absorbed in the prostate at a cellular level enabled the clinicians to determine the stage of the disease accurately and subsequently allowed for early detection of cancer recurrence, which improved patient outcomes. The semiquantitative analysis of PET/CT imaging with the use of the above-mentioned semiquantitative indices enables the quantitation of the burden of the disease. In addition, new studies correlate these imaging parameters with biochemical parameters like PSA (Figure 1).

Currently, the prostate cancer molecular imaging standardized evaluation criteria (PROMISE criteria) and the updated PROMISE V2 criteria have been proposed as a framework for whole-body staging to assess the prostate cancer disease extent on PSMA-PET. These criteria provide a common framework for defining quantifiable parameters in PSMA-PET that can assess treatment response or prognosis. The standardized reporting framework of PROMISE V2 is useful in a range of indications including staging of high-risk patients, biochemical recurrence and evaluation of suitability for 177Lu-PSMA radioligand therapy. The above-mentioned, evidence-based indications are now included in the clinical practice guidelines [50,51]. In the future, integrating serum and imaging biomarkers in the decision-making process may enable the optimization of our treatment approach tailored to each patient’s unique characteristics and semiquantitative analysis will remain a useful tool in prostate cancer staging.

## Figures and Tables

**Table 1:** Commonly used radiotracers for prostate cancer imaging.

		Basic Characteristics
PSMA based radiotracers	[^68^Ga]Ga-PSMA-11 [^18^F]DCFPyL, [^18^F]PSMA-1007, [^18^F]JK-PSMA-7, [^18^F]rhPSMA-7, [^18^F]AlF-PSMA-11	^68^Ga based radiotracers: on-site generator short half-life lower radiation exposure ^18^F-based radiotracers: higher image resolution, lower end-point positron energy, longer half-life, cheaper production, more false positives findings, greater locoregional lesion detection rate, accuracy in the delineation of the local lesion, greater lesion SUV uptake
FDG based radiotracers	^18^F-fluorodeoxyglucose	Prognostic biomarker in advanced prostate cancer use in PSMA-negative disease and in theragnostics approaches

**Table 2:** Overview of the basic characteristics of the included studies.

Authors (Year)	Number of Patients Included	Radiotracers	Semiquantitative PET/CT Parameters	Main Findings
Dong et al. (2022) [35]	60	^18^F-PSMA	SUVmax TL-PSMAp, PSMA-TVp	Semiquantitative analysis of the primary tumor on ^18^F-PSMA-1007 PET/CT imaging contributes to the risk stratification of prostate cancer
Yi et al. (2023) [36]	147	^68^Ga-PSMA	SUVmax	SUVmax is a predicting factor of intermediate and high-risk prostate cancer
Heetman et al. (2024) [37]	386	^68^Ga-PSMA	SUVmax	SUVmax ameliorates diagnostic accuracy in predicting the likelihood of clinically significant prostate cancer in biopsy material
Rogic et al. (2024) [38]	34	^68^Ga-PSMA	SUVmax	A positive correlation was found between intraprostatic SUVmax and ISUP group
Ali et al. (2023) [39]	154	^68^Ga-PSMA	SUVmax	SUVmax was both correlated with Gleason score and prostate-specific antigen (PSA) in organ-confined prostate cancer The median SUVmax and PSA directly related to Gleason score
Bela Andela et al. (2024) [40]	86	^68^Ga-PSMA	SUVmax ASP PSMA-TV PSMA-TLU	Significant association of PSMA-TV PSMA-TLU and ASP with overall survival (OS)
Bodar et al. (2022) [41]	318	^68^Ga-PSMA	SUVmax	SUVmax is correlated with with pISUP score and pathological tumor stage
Fragkiadaki et al. (2024) [33]	104	^18^F-PSMA	SUVmax TMTV	There is a positive correlation between PSA levels with the semiquantitative parameters SUVmax and TMTV of the metastatic foci
Cardoza-Ochoa et al. (2022) [42]	110	^18^F-PSMA	wbTl-PSMA wbPSMA-TV	Statistically significant correlation of between wbTL-PSMA and wbPSMA-TV with serum PSA.
Li, Y. et al. (2024) [43]	110	^18^F-PSMA	TL-PSMAp PSMA-TVp SUVmax	TL-PSMAp and PSMA-TVp can distinguish between oligometastatic and extensive metastatic disease and can predict oligometastatic disease

SUVmax: standard uptake value maximum TL-PSMAp: prostate total lesion prostate specific membrane antigen, PSMA-TVp: prostate specific membrane antigen-tumor volume, PSMA-TV: prostate specific membrane antigen receptor-expressing tumor volume, PSMA-TLU: prostate specific membrane antigen total lesion uptake, wbTl-PSMA: whole-body total lesions prostate specific membrane antigen uptake, wbPSMA-TV: whole-body prostate specific membrane antigen tumor volume.

## Abbreviations

PET/CT	Positron emission tomography/computed tomography
^68^Ga-PSMA	^68^Gallium-prostate-specific membrane antigen
[^18^F]-FDG	^18^F-fluorodeoxyglucose
SUV	Standard Uptake Value
SUVmax	Standard Uptake Value maximum
MTV	Metabolic tumor volume
TMTV	Total metabolic tumor volume
PSMA	TV PSMA Total Volume
PSMA TLU	Psma total lesion uptake
TL-PSMA	Total lesion PSMA
PSMA-TVp	Prostate PSMA-tumor volume
ASP	Tumor asphericity
wbPSMA-TV	Whole-body PSMA tumor volume
wbTL-PSMA	Whole-body total lesions PSMA uptake
OS	Overall Survival
SUVmean	Mean standard uptake value
SUVpeak	Peak standard uptake value
SUL	Lean standard uptake value
SUVbsa	Body surface area standard uptake value
AUC	Area under the curve
